# Can Dogs Limbo? Dogs’ Perception of Affordances for Negotiating an Opening

**DOI:** 10.3390/ani11030620

**Published:** 2021-02-26

**Authors:** Alexandra Horowitz, Eloise West, Molly Ball, Blakeley Bagwell

**Affiliations:** Dog Cognition Lab., Department of Psychology, Barnard College, New York, NY 10027, USA; ewest@psych.ubc.ca (E.W.); miball@me.com (M.B.); bmb2192@barnard.edu (B.B.)

**Keywords:** domestic dogs, affordances, behavior, body size, size sense, size perception, sense of self, animal cognition, self-representation

## Abstract

**Simple Summary:**

Recent behavioral research with domestic dogs has focused largely on their social cognition: how they interact with and interpret both other dogs and humans. Less well studied are the various aspects of their perceptual experience which might provide knowledge of how they understand the non-social world and themselves. In two studies, we look at how dogs navigate their environment. We first set up a situation to test whether dogs understand when they are too big to go through an opening; we also look at how they adjust their bodies to increasingly smaller (shorter) openings. We then also look at how dogs navigate an opening when their body width is effectively increased by their holding a stick in their mouth. We find that dogs show more hesitation approaching openings that are too small than ones through which they comfortably fit. Dogs of all sizes also change their behavior in a uniform way to negotiate short openings. When holding a stick, dogs did not initially change their behavior but are able to negotiate through an opening with experience. Researching how dogs navigate through a changing environment may be a fruitful way to begin to understand their sense of themselves.

**Abstract:**

Very little research has focused on canines’ understanding of their own size, and their ability to apply this understanding to their surroundings. The current study tests domestic dogs’ judgment of their body size in relation to a changing environment in two novel experimental situations: when encountering an opening of decreasing height (Study 1) and when negotiating an opening when carrying a stick in their mouth (Study 2). We hypothesized that if dogs understand their own body size, they will accurately judge when an opening is too small for their body to fit through, showing longer latencies to approach the smaller openings and adjusting their body appropriately to get through—although this judgment may not extend to when their body size is effectively increased. In line with these hypotheses, we found that the latency for subjects to reach an aperture they could easily fit through was significantly shorter than to one which was almost too small to fit through. We also found that the order of subjects’ adjustments to negotiate an aperture was invariant across individuals, indicating that dogs’ perception of affordances to fit through an aperture is action-scaled. Preliminary results suggest that dogs’ approach behavior is different when a horizontal appendage is introduced, but that dogs were able to alter their behavior with experience. These results are consistent with the hypothesis that dogs understand their own body size and the affordances of their changing environment.

## 1. Introduction

Research since the late 1990s has substantially broadened our understanding of the cognitive abilities of the domestic dog—in particular, how dogs perceive the world and interact with others. The greatest majority of research focuses on social cognitive abilities, such as gaze- and point-following [1,2] and use of others’ attention [3,4]; other research looks at the possible result of such social cognitive skill, such as perspective-taking [5,6] and representation of self [7]. Less well studied are the contributions of foundational perceptual abilities to cognition.

Recent work has begun to investigate the cognitive consequences of olfactory perception [8,9]; very few research programs have examined perception of affordances and body-size awareness [10,11,12]. Animals’ information about their body size, especially relative to environmental changes and constraints—body sense—is critical to navigating new environments as well as social success. An animal’s size guides their behavior and, for many species, can be a key determinant in both their short- and long-term survival [13,14]. Relative body size also relates to reproductive output [15,16] and fighting behavior [17]. A size sense is also integral to navigating changing environments: it has been proposed that the capacity for self-concept evolved to enable large animals to navigate complex environments [18]. While most animals’ environments fluctuate with naturalistic changes, such as a fallen tree blocking a habitual route, the environments of species who live among humans are additionally driven by human behavior. For an owned dog living in a human household, various aspects of their environment may be altered daily: a chair pushed away from a table or a door closed partway. In these scenarios, an animal’s sense of their own size would facilitate negotiation of their changing surroundings [19,20].

Previous research with dogs has investigated their perception of size primarily of conspecifics, not themselves. In a looking-time paradigm, Taylor et al. [21] found that dogs could match growls of different pitches with corresponding images of dogs of different sizes. Other work has found that dogs accurately matched food-guarding growls with an image of the source of the growl, but looked toward the image of a dog larger than the source when hearing playful growls [22]. Some research addresses a dog’s understanding of their body size with respect to the size of other dogs. Dogs self-handicap with smaller dogs in play [23] and small dogs perform higher raised-leg displays relative to their body size than large dogs do [24].

Having an understanding of one’s size relative to features in the environment also enables determination of what actions are possible in a given scenario, or the “affordances” of the environment [20,25]. Human studies show that perceptual input—primarily visual—is used to guide decisions about what actions can be performed [20], sensitive to changes of one’s own body and of one’s environment [26]. Subjects attempt to reach or move through openings larger, but not smaller, than their appendage or body size, and make appropriate adjustments to move through narrow openings [27,28]. Moreover, subjects wearing size-increasing prosthetics (such as of the hand or stomach) made appropriate adjustments, with experience [27,29]. In research with canine subjects, Wagman et al. [12] evaluated dog behavior when attempting to acquire a treat placed at increasingly higher points along a wall; they found that the height at which subjects transition from reaching for a treat with their head only to rearing was different across individuals but with the same ratio of shoulder height to treat height [30]. Two projects looked at dogs’ behavior when approaching differently sized openings. Maeda and Fujita [11] found that when simultaneously given a larger or smaller doorway opening, subjects preferred to go through the larger opening. Lenkei et al. [10] found longer latencies for subjects to approach too narrow or short openings than openings through which they could fit; moreover, subjects did not attempt to pass through openings that were too small [10]. These studies provide preliminary evidence that dogs can perceive affordances of a given environment and integrate information about the external world and their own body to organize their behavior.

The present research extends this literature into dogs’ knowledge of their body size, and further explores their understanding of the affordances of the environment. In the first study, we assess whether dogs understand their size relative to an opening which they are asked to pass through by examining behavioral modifications and approach time as the opening becomes increasingly shorter. As in Lenkei et al. [10], we assess subjects’ judgment of their ability to fit through the openings by measuring their latency to approach openings of decreasing height. We predict that if dogs have a knowledge of their own body size, then they will accurately judge which openings are passable, showing longer latencies to approach smaller openings. In addition, we analyze dogs’ physical adjustments as they attempt to pass through increasingly smaller openings, and examine how their behavior relates to their height at withers [12], eye-height [31], or elbow joint-height (which may differ by breed [32]). As the opening is adjusted incrementally along one dimension, we can determine the ratio at which dogs first adjust their behavior to negotiate the opening, relative to subjects’ height at withers. We predict that the order of physical adjustments will be invariant across dogs, and thus consistent across different body sizes, as will the ratio of body-height to the height of the opening at which dogs make their first bodily adjustment.

Given the importance of tactile information, acquired via experience, to subject behavior [29], in a second study, we examine subject behavior when their body size is effectively increased. To naturalistically and temporarily increase their width, we offer dogs sticks of various lengths to hold in their mouths while passing through a fixed-width opening. We measure subject modifications in approach time and body position with variously sized sticks, and offer subjects the chance to choose a stick that will easily fit through the aperture. We predict subjects will inaccurately judge affordances as stick length increases—leading to an inability to fit through the opening—but will be able to update their perception of possible actions with experience, and alter their approach so to fit.

## 2. Materials and Methods

### 2.1. Participants

Dogs and owners were recruited through the Barnard Dog Cognition Lab database. Forty-four domestic dogs (23M, 21F) and their owners began the study. Subject dogs were required to be at least 8 months old, healthy, fully vaccinated, and comfortable with new people and in new environments. The mean subject age was 5.5 years (range: 1 year 4 months to 11 years 4 months); all but 2 dogs were spayed or neutered. Twenty-five dogs were described by their owners as purebred and 19 were mixed breeds (Table 1).

### 2.2. Design

Prior to participating, owners completed a questionnaire about their dog’s breed, age, health, size, and training history, as well as behavioral characteristics like temperament, ability to follow commands, food motivation, and stick-carrying behavior. Based on owners’ measurement of their dogs’ height at withers, subjects were assigned to a category from XS to XL (see Table 1) in order to determine aperture heights in Study 1; this height was confirmed when the subject arrived at the lab. Dogs described by their owners as both habitually “carrying sticks,” as well as engaging in two or more other stick-related behaviors (“retrieves stick after it’s thrown”; “keeps sticks at home”; “has a favorite stick”; “presents a stick to you to throw or tug on”; “chews stick”) were also invited to participate in Study 2. Owners brought their dogs to the Dog Cognition Lab on Barnard College’s New York City campus, completed a consent form, and were informed about their role in the studies, outlined below. Owners with a dog in Study 2 additionally brought a favored dog toy from home. Participants were scheduled to arrive in sequence, so that only one dog was present in the room for each trial. Testing took place from January 2020 to March 2020.

### 2.3. Apparatus and Stimuli

**Figure 1:**
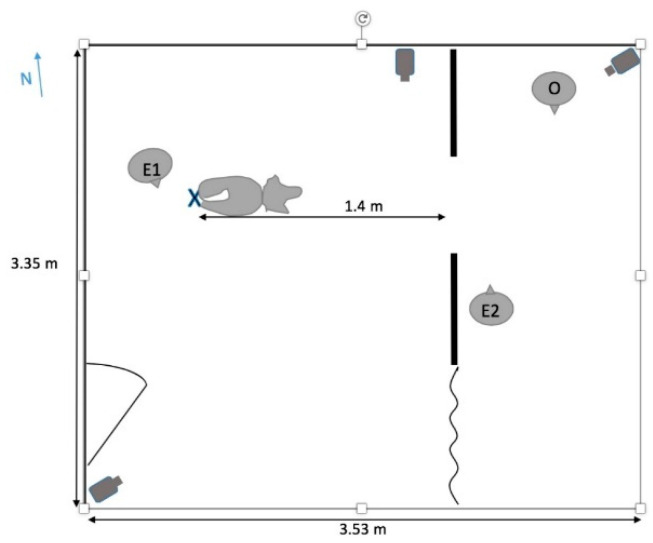
Layout of Dog Cognition Lab with position of subject, apparatus, experimenters (E1 and E2), owner (O), cameras, and magnetic north (N) indicated.

The testing room at the Barnard Dog Cognition Lab is 3.53 × 3.35 m, with a single door and no windows. Three cameras (Lorex 1080p, Lorex, Markham, ON, Canada) are situated to record trials: two capturing room views, from the northeast and southwest corners of the room; a third low on the northern wall, pointing toward the experimental theater (Figure 1).

**Figure 2:**
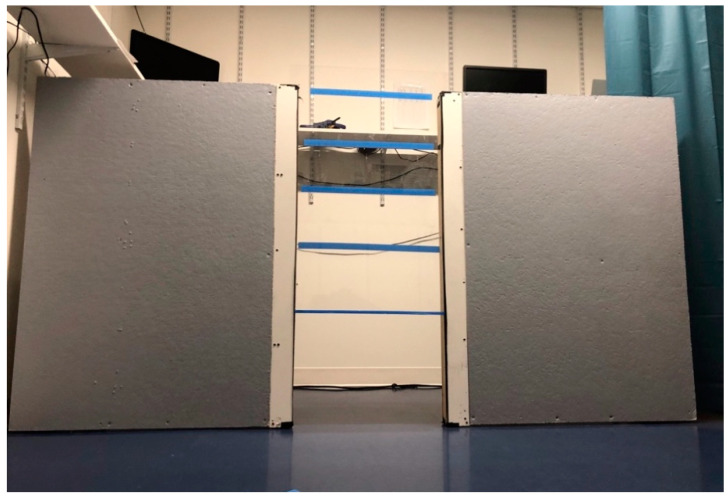
Experimental apparatus from the subject’s point of view, with aperture and Plexiglas (marked with blue tape) within it.

The experimental apparatus consisted of two upright wooden members serving as walls of 1.8 m width and 1.2 m height around an opening (hereinafter “aperture”) of width 61 cm (Figure 2). The edges of the walls around the aperture were fitted with tracks that allowed a Plexiglas panel to be raised or lowered, enabling experimenters to adjust the height of the aperture to specific increments relative to the dog’s height at withers. On one side of the apparatus, a curtain was pulled across a second opening serving as an indirect, alternative route around the apparatus. The alternate route was available so that subjects did not become overly frustrated if they could not reach their owners. The owner sat in a chair behind the apparatus, while the dog was held on a short leash 1.4 m in front of the apparatus.

A transparent Plexiglas was used to enable the dogs to see their owners seated on the other side of the apparatus; blue painter’s tape was applied to the Plexiglas panel in order to make it more visible (no subjects ran into the barrier).

For Study 2, sticks of three lengths—small enough to fit in (46 cm), just the size of (61 cm), or too large to fit in (76 cm) the aperture—were collected from Riverside Park in New York City.

To control for unintended odors, the floor was cleaned with a solution of 70% isopropyl alcohol between subjects. The temperature of the room was recorded at the beginning of trials, and varied from 70.1 to 79 degrees Fahrenheit (mean = 74); humidity levels ranged from 11 to 34% (mean = 24%).

### 2.4. Experimental Procedure

An experimenter met the owner and dog at a street-level entrance to the College and walked them upstairs to the testing space. Upon arrival in the lab, the owner was asked to remove their dog’s leash and the dog was allowed to explore the room independently and become acclimated to the testing space while the experimenters explained the owner’s role in the studies. When the dog appeared calm, the owner was given a short leash to attach to their dog’s collar. While the owner held the dog’s leash, experimenters measured the height of each dog at their withers, eyes, and front elbows.

#### 2.4.1. Study 1: Size Sense

*Warm-up trial*. The owner was seated behind the experimental apparatus; the first experimenter (E1) sat on the opposite side of the apparatus from the owner, holding the dog by the leash. A second experimenter (E2) stood behind the apparatus to move the Plexiglas to adjust the aperture size (height of the opening) in each trial. In order to be as neutral as possible, experimenters avoided staring at, talking to, or petting the subjects.

The Plexiglas was removed so that the aperture had no upper limit. The owner was given a treat for their dog, and E2 announced the start of the trial by asking the owner to call their dog by saying “[Name], come here!” or as they usually did. When their dog passed through the aperture, the owner gave them the treat. This procedure was repeated until a trial was successfully completed (the dog passed through the aperture). The experiment was aborted if the dog did not pass through the aperture after five attempts (only 1 dog did not pass through the aperture in five attempts).

*Test trials*. The experimental set-up was identical to the warm-up trial, except that the Plexiglas panel was slid downward in the tracks to create the upper edge of the aperture at specific increments relative to the subjects’ height at withers (HW; or an averaged size, should the dog not cooperate with being measured (*n* = 1)). Dogs’ bin category (from XS to XL) was used to determine the specific aperture sizes on the first trial and second trial, and the increments by which the Plexiglas panel were lowered, to make consistently smaller apertures, for subsequent trials (Table 2).

When E2 announced the start of the trial, the owner, seated behind the apparatus, called their dog by saying “[Name], come here!” or as they usually did. The owner gave their dog a treat after the dog reached their side of the apparatus, regardless of whether they passed through the aperture or arrived via an alternative method. After completing each trial, E1 retrieved the dog and brought them back to the starting position. E2 then lowered the Plexiglas panel by the predetermined increment to make an aperture of the designated height. This procedure was repeated until the dog refused to pass through the aperture or pursued an alternate route and walked behind the curtain. Following refusal or pursuit of an alternate route, the trial was repeated once with the same sized aperture. If the dog went through the aperture on this trial, the experiment resumed and the Plexiglas panel was lowered once more. If the dog did not go through the aperture on the repeated trial, the experiment was concluded.

#### 2.4.2. Study 2: Stick-Carrying

Following Study 1, those dogs (*n* = 13) described by their owners as stick-carrying dogs (see Design, Section 2.2, above) participated in Study 2. They were allowed to rest or play off leash as E2 explained the next study’s procedure.

*Warm-up trial*. The starting positions for the subject, owner, and apparatus were the same as those in Study 1, except that the aperture was completely open (i.e., had no height ceiling) throughout the trials. As in Study 1, the aperture width was constant (at 61 cm) through the trials. E1 gave the dog a toy that the owner had brought from home. When the dog was holding the toy, E2 announced the start of the trial. The owner called the dog through the aperture using their typical request (such as “[Name], come here!”). If the dog passed entirely through the aperture while still holding the toy, the owner gave the dog a treat. E1 then brought the dog to the starting position. This procedure was repeated until the dog completed two warm-up trials successfully. If the dog did not pass through the aperture while holding the toy across five attempts, the experiment was concluded.

*Test trials*. With the aperture width held constant, the dogs were offered sticks of three different sizes (i.e., when held horizontally, three different widths). E1 offered the dog the 46 cm stick. When the dog was holding the stick in their mouth, E2 announced the start of the trial. The owner called their dog and rewarded them if and when they passed all the way through the aperture while still holding the stick. E1 then returned the dog to the starting position. The same procedure was followed with the 61 cm stick and then with the 76 cm stick. At each stage, the trial was repeated until the dog successfully passed through the aperture, refused to pass through the aperture, dropped and abandoned the stick, or pursued an alternate route. In the latter three cases, the trial was repeated once with the same sized stick. If the dog successfully completed the repeated trial, the sequence was resumed. If they again refused to pass, dropped the stick, or pursued an alternate route, the experiment was concluded.

Following the final trial, E1 returned the dog to the starting position and placed all three sticks on the ground in front of the dog. E1 then asked, “What’s this?” If the dog picked up a stick, E1 noted the stick size. The owner called the dog through the aperture and gave their dog a treat if and when the dog passed through the aperture completely.

### 2.5. Behavioral Coding

**Figure 3:**
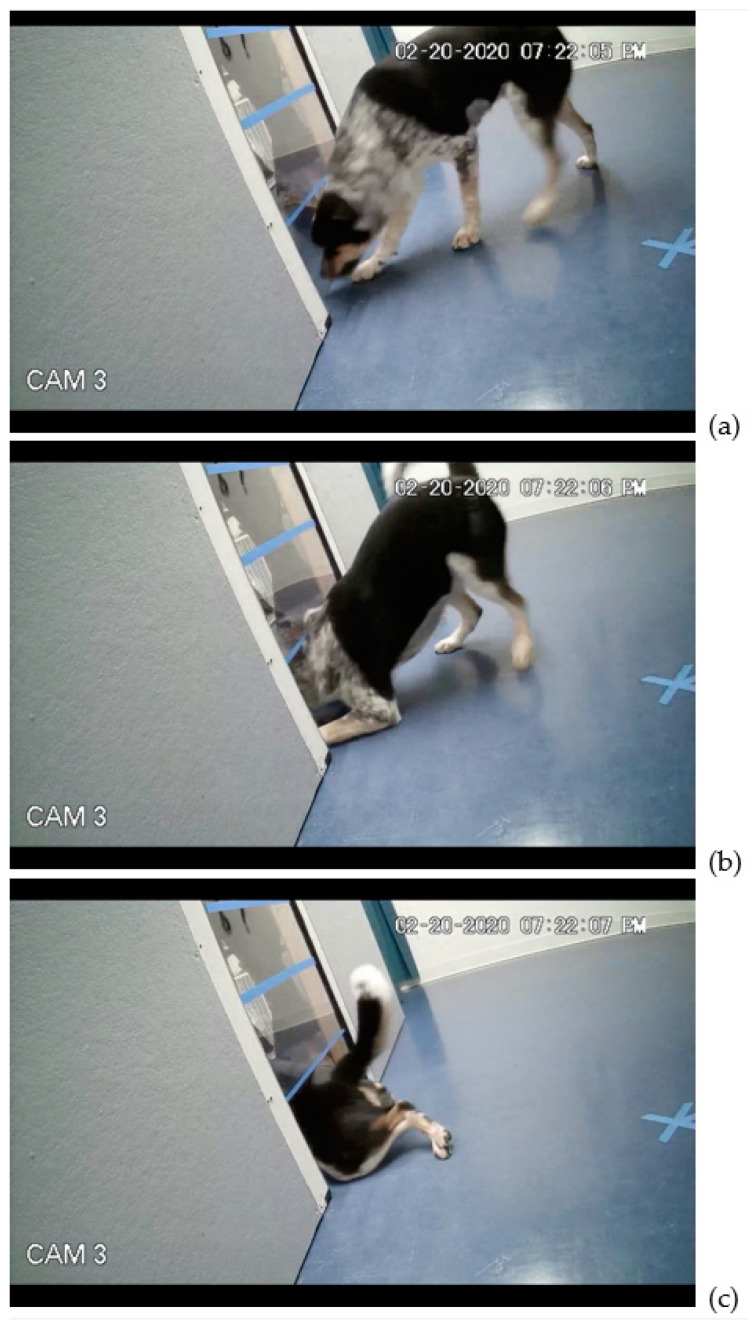
Example of head duck (**a**), front-elbow bend (**b**), and back-elbow bend (**c**).

Video cameras captured all subject behavior in both studies for later frame-by-frame playback (30 fps) and coding by one of the authors (BB). On each trial, we calculated the subject’s latency to reach the aperture—the time from E1’s release of the dog to the dog’s arrival at the opening—as well as the order of behavioral modifications made by the subject in negotiating the aperture: head duck, head or body turn, front elbow bend, rear elbow bend, and alternate routes taken (Figure 3). We also coded each subject’s pass-through successes and pass-through attempts on each trial, and noted the point to which subjects get through the aperture on aborted and unsuccessful attempts. Additionally, in Study 2, we noted the number of head and body rotations; number of times the subject knocked the side of the aperture with the stick; and stick choice on final trial, if any. For Study 1, subjects were divided into two height groups post hoc, per previous height binning [12]: short (height at withers less than 54 cm) and tall (height at withers greater than or equal to 54 cm). We applied a linear regression analysis for latencies to reach the aperture, chi-squared goodness-of-fit tests for order of modifications, and independent-sample t-tests to compare subjects’ smallest aperture by bin and to calculate ratios of dog height to height of aperture at first adjustment. Reliability of coding was gauged against a second, independent coder of the videos. Inter-observer agreement was high for latency to reach the aperture (*n* = 118 trials; Spearman’s rho = 0.51, *p* < 0.001) and for the first bodily adjustment on (*n* = 11 subjects, Cohen’s Kappa = 0.76).

## 3. Results

### 3.1. Study 1

Forty-three dogs participated in 424 trials (*n* = 1 dog refused to participate in any of the trials). Subjects were categorized into five size bins on the basis of height at withers: XS (*n* = 5), S (*n* = 9), M (*n* = 13), L (*n* = 16), XL (*n* = 1) (see Table 2 for height ranges per category, and Table 1 for individual participant characteristics). For analysis, the single XL dog was included in the L bin category.

**Figure 4:**
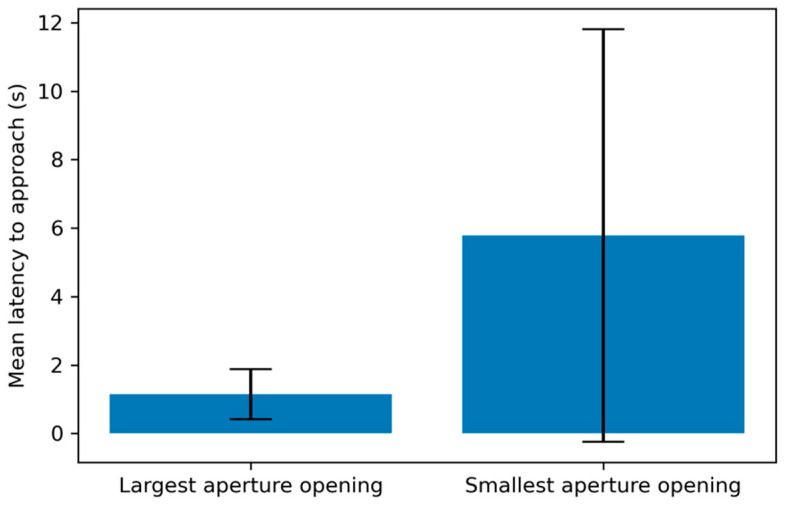
Mean latency for subjects to reach the aperture at its largest opening (first trial) and at its smallest opening (final trial with successful passage) (*p* < 0.001). Subjects took significantly longer to reach the aperture at the final trial than at the first trial. Error bars represent standard deviation.

Subjects attempted to pass through the aperture an average of 9.65 times (SD = 2.14), and successfully passed through the aperture an average of 7.57 times (SD = 1.98). A linear regression of subjects’ age and the number of successful trials completed showed no significant correlation between age and performance (*r*^2^ = 0.01, *p* > 0.05). The subjects modified their behavior on an average of 6.09 trials (SD = 1.76), or an average of 61.8% of all the trials they completed (SD = 15.6%). Subject latency to reach the aperture depended on its height: latency was significantly longer on subjects’ final successful trial (M = 5.78 s, SD = 6.02) than on the first trial (M = 1.15 s, SD = 0.73) t(42) = −4.95, *p* < 0.001) (Figure 4). There was no significant difference in latency between the first trial and the first trial with a behavioral modification (t(42) = −1.40, *p* = 0.16).

**Figure 5:**
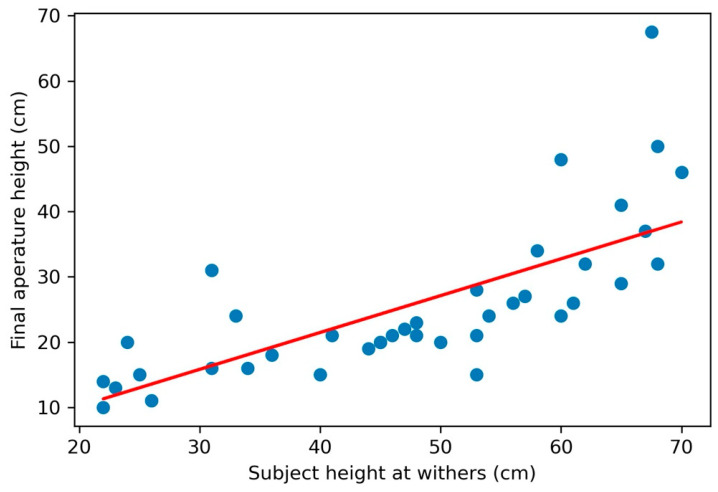
The relationship between subjects’ final aperture height and height at withers (HW). A significant positive correlation was found between the final aperture height subjects successfully passed through and subjects’ height at withers (*p* < 0.001).

Across subjects, the final aperture height negotiated varied according to the subjects’ size. A linear regression of subjects’ height at withers and the aperture height on the final successful trial—the trial on which the subject passed through the aperture—showed a positive correlation (*r*^2^ = 0.52, *p* < 0.001; Figure 5). Eye height (*r*^2^ = 0.44, *p* < 0.001) and elbow height (*r*^2^ = 0.50, *p* < 0.001) were also positively correlated, less strongly, with final aperture height.

**Figure 6:**
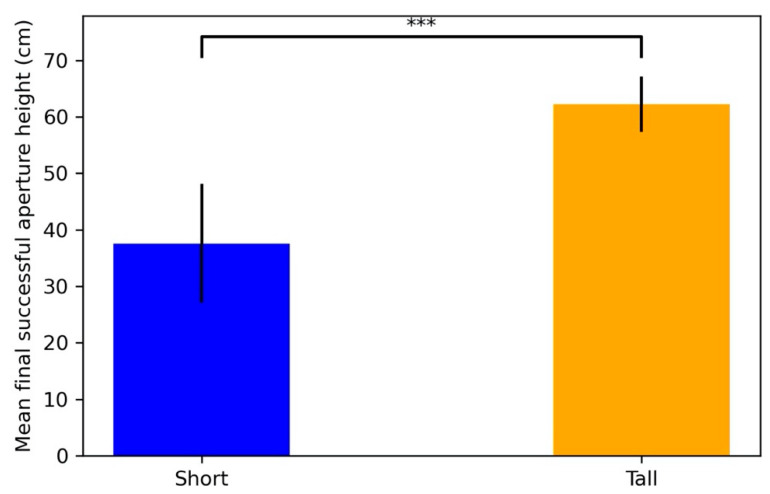
Mean height of aperture at final successful trial for short and tall dogs (*** *p* *≤* 0.001).

The final aperture height successfully negotiated was significantly higher for dogs in the tall group (*M* = 62.22 cm, SD = 4.90) than for dogs in the short group (*M* = 37.58 cm, SD = 10.54), *t*(41) = −8.57, *p* < 0.001; Figure 6). Considered by their bin category, the final aperture height successfully negotiated was significantly higher for dogs in the large group (M = 35.66 cm, SD = 11.70) than for dogs in the medium group (M = 20.5 cm, SD = 3.15), t(41) = −4.40, *p* < 0.001), the small group (M = 19.0 cm, SD = 7.42), t(41) = −3.70, *p* = 0.001), and the extra-small group (M = 14.4 cm, SD = 3.26), t(41) = −3.82, *p* = 0.001). Furthermore, the final aperture height was significantly higher for dogs in the medium group than dogs in the extra-small group (t(41) = −3.41, *p* = 0.003).

To determine if the height at which dogs made their first adjustment was the same across dogs of different size, we calculated the mean aperture height for the first adjustment, divided by dog’s HW, for both short and tall dogs. Comparison of these ratios found no significant difference for dog sizes (*M*short = 0.91; *M*tall = 0.91; t(41) = −0.07, *p* > 0.05).

To examine whether dogs used a common strategy to negotiate the aperture, Chi-squared goodness of fit tests were computed on frequencies of the use of specific behavioral adjustments to fit through the aperture—as well as the frequencies of overall adjustment sequences across trials. Subjects’ first behavioral adjustment was significantly more likely to be a head duck than any of the other behaviors (*X^2^*(3, *n* = 43) = 97.05, *p* < 0.001). The second adjustment was often either a front-elbow bend or a back-elbow bend (*X^2^*(3, *n* = 43) = 34.02, *p* < 0.001), with no significant difference between the two (*X^2^*(1, *n* = 39) = 0.02, *p* = 0.63). Similarly, the third behavior was most often either a front elbow bend or back elbow bend (*X^2^*(3, *n* = 43) = 28.56, *p* < 0.001), with no significant difference in frequency between the two (*X^2^*(1, *n* = 37) = 0.68, *p* = 0.41). The fourth behavioral adjustment was significantly more likely to be the body turn (*X^2^*(3, *n* = 43) = 39.78, *p* < 0.001) than any other behavior.

**Figure 7:**
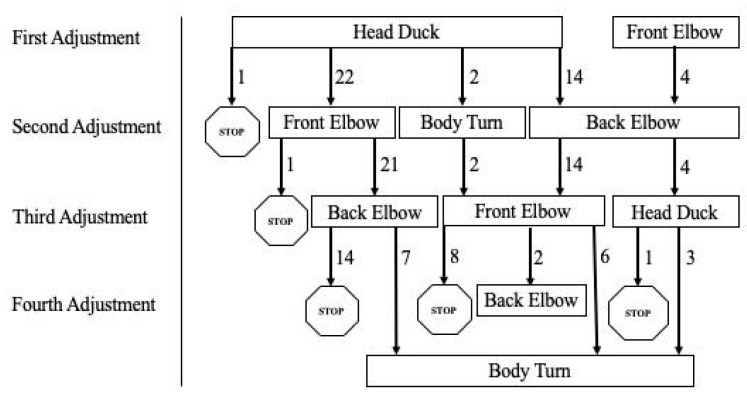
Transition diagram of behavioral adjustments used by all subjects to negotiate the aperture. Order of adjustments goes from top downward, with arrows indicating the number of subjects who followed each sequence to either another adjustment or to a stop (no further adjustment).

Moreover, adjustment sequence “head duck–front elbow–back elbow–body turn” and “head duck–back elbow–front elbow–body turn” were significantly more likely to occur than any other order of behavioral adjustments (*X^2^*(3, *n* = 41) = 23.10, *p* < 0.001) (Figure 7). There was no significant difference between the frequency of these two sequences. An independent Chi-square test revealed no significant relationship between the adjustment sequence chosen and subject body size (*X^2^*(16, *n* = 41) = 7.94, *p* = 0.54).

**Figure 8:**
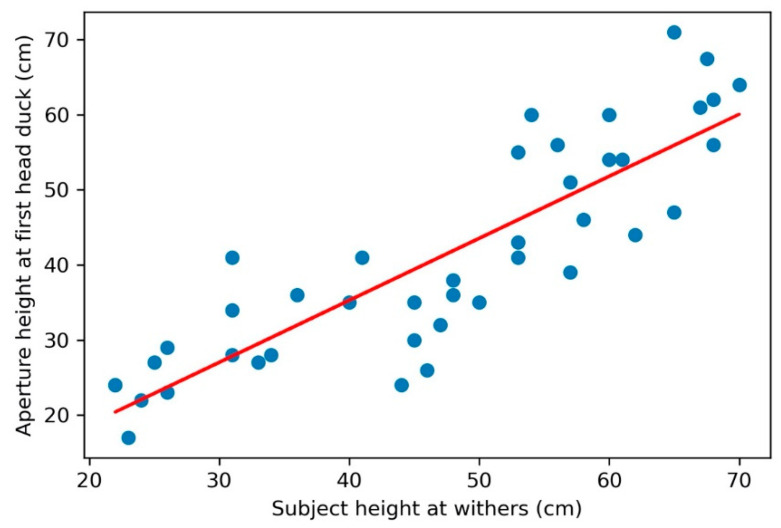
The relationship between the aperture height at subjects’ first head adjustment and subjects’ height at withers (HW). A significant positive correlation was found between the aperture height the first time subjects ducked their head to pass through and subjects’ height at withers (*p* < 0.001).

**Figure 9:**
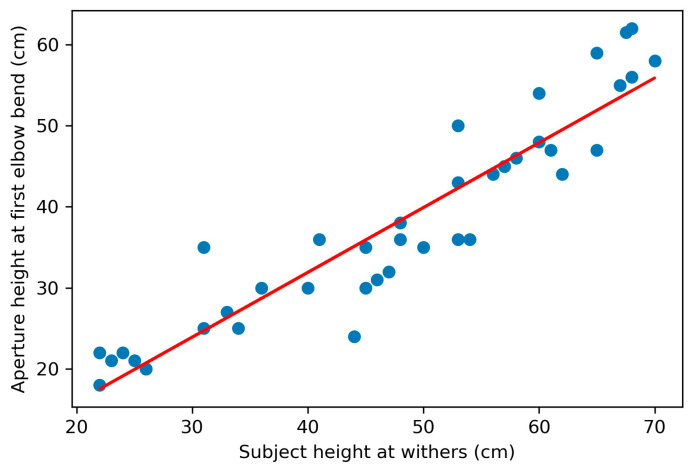
The relationship between the aperture height at subjects’ first elbow adjustment and subjects’ height at withers (HW). A significant positive correlation was found between the aperture height the first time subjects bent their elbows to pass through and subjects’ height at withers (*p* < 0.001).

A linear regression of subjects’ height at withers on the aperture height when subjects made specific behavioral adjustments revealed a strong positive correlation with height at head duck, with height at withers accounting for 75% of variance in the height of aperture (r^2^ = 0.75, *p* < 0.001) (Figure 8). A significant correlation is also seen between height at withers and aperture height at which subjects first bent their elbows (either front or back) (r^2^ = 0.87, *p* < 0.001) (Figure 9).

Three dogs used the alternate route on one trial: two on their final height, after failing to go through the aperture, and one who used it but later returned to navigating the opening on the next trial.

### 3.2. Study 2

Thirteen dogs were selected to participate in Study 2; three dogs did not successfully complete the training trials, and four dogs did not successfully complete the first experimental trial. Thus, data were analyzed from *n* = 6 dogs. Four of the six dogs fully completed the procedure: one did not make it through the aperture successfully on all trials.

Due to the high attrition rate, inferential statistical analysis was not conducted; all analyses are descriptive. All six subjects successfully passed through the aperture with the small and large stick; all but one successfully passed through with the medium stick. Subjects attempted to pass through the aperture an average of 1.5 times with the small stick, 1.17 times with the medium stick, and 1.67 times with the large stick.

Latency to reach the aperture with the small stick was 21.79 s (SD = 27.58), excluding one subject outlier who had an approach time of 1020 s. Latency to approach with the medium stick was 1.54 s (SD = 0.76); with the large stick, 4.06 s (SD = 5.21).

Behavioral adjustments on these trials included head turns (small stick: 1; medium stick: 1; large stick, 2) and dropping the stick (mean for small stick: 1; medium stick: 0.83; large stick, 1.16). Subjects more often hit the side of the aperture with the large stick (mean = 1.33) than with the small or medium sized sticks (mean = 0.5). When all six subjects were given a final choice of stick on the final trial, three chose the large stick, one chose the small, one chose the medium, and one made no choice.

## 4. Discussion

These studies were designed to address subject dogs’ behavior when asked to go through an opening (aperture) of decreasing height, until they no longer fit through the opening. The results speak to dogs’ perception of their body size with respect to their environment. This suggests that dogs not only perceive others’ body sizes, as previously found [21,22,24], but also their own. The dogs in this study used knowledge of their body size when judging whether they would fit through an opening: the latency for subjects to reach an aperture they could easily fit through was significantly shorter than to one which was almost too small to fit through. This finding is consistent with Lenkei et al. [10], who also found a longer latency to approach a too-small opening. Both our results suggest that dogs distinguish between easily passable and difficult to pass openings before acting.

As expected, the final height of the aperture differed significantly between short and tall dogs and across size bins (XS, S, M, L) and correlated with several measurements: the dog’s height at withers, elbows, and eyes. This indicates that what is considered “too small” to fit through is dependent on the dog’s sense of their own size. Moreover, as all measurements of the dog’s body appear to influence their perception of affordances, it is possible that the dog uses a crude representation of their body to determine what actions are possible. Notably, at the same time, subjects’ increase in latency across trials cannot be explained by, for instance, a decrease in motivation as the aperture diminished in height, as most subjects continued to attempt to squeeze through the opening even when it was impossible for them to pass through it.

Interestingly, in the present study, 12 subjects passed through an aperture shorter than one-third of their height at withers; this is smaller in height than what was considered “too small” in Lenkei et al. [10]. Many more subjects unsuccessfully attempted to pass through an opening that was in fact too small for them to fit through even with bodily modifications. This difference between subject behavior in the present study and in Lenkei et al. [10] might be explained as resulting from the latter’s adjustment of both the height and width of the aperture (in their Experiment 2), while the present study only adjusted the height. An aperture which is not wide enough to pass through may be more obviously “too small”, as subjects cannot adjust their behavior to significantly reduce their width. By contrast, dogs can adjust their height by, for example, ducking their head or bending their elbows. In addition, decreasing the aperture size incrementally may have enabled more precision in determination of what size was too small for the subjects.

Additionally, the subjects’ different behavior may be related to the cost or benefit associated with negotiating the experimental apparatus in the two studies. Studies with human infants have found that subjects decide whether to use a small doorway according to the related risk: many infants err and get stuck when squeezing into too small openings, but they do not attempt a too small aperture when such an error would result in falling [33]. This indicates that infants can perceive the affordances allotted by an aperture, but also use information about the cost of using the aperture when planning their actions. Dogs may be similarly evaluating the associated risk during their decision-making process: there was very little risk in passing through the similar apparatuses used in Lenkei et al. [10] and in this study. However, in addition, in the present study, there was an added benefit: their owners held a treat on the opposite side of the apparatus and were reachable only by successfully negotiating the apparatus. While Lenkei et al. [10] suggested that dogs’ motivation to get through their opening was high even without the owners (Experiment 1) or without a treat (Experiment 2 and 3), the present study points to the added motivation present when both owner and treat are reached by completing the task.

To further understand the dog’s decision-making process, we sought to determine not only when a dog decides whether they can or cannot fit through an aperture, but also *how* dogs fit through an aperture. Thus, we extended our analyses to the order of behavioral adjustments both across and within trials. Through examination of the sequence of bodily modifications made by subjects as they negotiated ever-smaller openings, we can suggest that subjects’ perception of affordances to fit through an aperture is action-scaled. Our results revealed that subjects’ first adjustment came at the same ratio of dogs’ height to aperture height across sizes. Moreover, we found a preferred sequence to adjustments: subjects most often first ducked their heads, then bent front or back elbows, then turned their body. This sequence was relatively invariant across individuals, regardless of body size, providing evidence that dogs know which actions are necessary in the face of environmental constraints. Across subjects, there was a positive correlation between the size of the aperture opening and the size of the subjects’ withers, eye-height, and elbow height. Dog behavior reflects an appreciation of the task—navigating the aperture—relative to the capabilities and constraints of their own bodies. The timing of specific bodily adjustments correlated with subject size: dogs’ height at withers predicts when subjects would adjust their bodies by ducking their head or bending their elbow to fit through ever-diminishing apertures. Similarly, Wagman et al. [12] found that dogs’ perception of the affordances for reaching was action-scaled, with the ratio of the stimulus height to body height at which subjects moved from reaching to rearing the same for tall dogs and small dogs. The present work adds to the small body of literature examining non-human animals’ navigation of novel environments, which has found, for instance, that snakes change behavioral strategies depending on the affordances of their environment [34], and frogs prefer jumping through horizontal openings than same-size vertical openings, in line with their physical profile [35].

Our results in Study 2 must be considered preliminary because of the small sample size. Indeed, subject data provide no clear message. Subject latencies to reach the aperture were longer with a stick that fit than with a stick that did not fit, against expectations. Subjects made multiple attempts to pass through the aperture with all stick sizes. In line with our prediction that subjects may inaccurately judge affordances when their bodies were effectively vertically extended, the sticks often hit the side of the aperture—more with the large-sized stick than the small- or medium-sized sticks. Certainly, subject behavior was not consistent with the idea that they had foreknowledge of the way the stick had changed their size. Similarly, it has been reported that dogs often bump into objects when navigating a familiar environment with an unfamiliar neck appendage like the Elizabethan collar, a flexible cone usually of plastic that is secured around a dog’s neck after surgeries in order to prevent the dog’s oral manipulation of a surgical site. This is suggestive that dogs’ perception of their size does not extend to appendages adding to their height or width.

Instead, modifications to their body (by turning their head) were performed only after knocking the apparatus: possible evidence that subjects updated their perception of affordances with experience. Future research examining these questions may want to include training for dogs to reliably hold or carry sticks in order to assure a robust sample size.

These results indicate that, much like humans [26,28], dogs integrate complex information about their body size with knowledge of how adjustment behaviors can alter their size when determining which actions are possible. Future research may explore the role of specific experiences in developing this “size sense”. Humans require experience with their artificially altered body size to update their perception of affordances [29], and preliminary evidence from Experiment 2 suggests that dogs are, at least initially, similarly inaccurate at determining affordances when their width is effectively increased.

This work suggests that dogs’ representation of their body size extends beyond the olfactory modality [8] and past actions [7]. Prior research on self-representation, using the mirror-mark test, has found mixed results with non-humans, some of which may be due to problems resulting from adapting primate-centric cognitive paradigms to non-primates [8]. Insofar as a sense of one’s size may be described as a representation of oneself [10,18], testing an animal’s sense of their size in a changing environment may be a novel methodological way to investigate the notoriously elusive sense of self.

## 5. Conclusions

Subject dogs demonstrate an understanding of their size via their differential latencies to approach openings of various sizes. This representation may not update when their size is temporarily increased by holding an object wider than their body width. Dogs’ perceptions of the affordances of this novel environment were action-scaled: subjects’ body size correlated with their bodily adjustments to the shortening aperture. Examining subject behavior in a novel environment may be a fruitful way to investigate self-representation in non-human animals.

## Figures and Tables

**Table 1:** All subjects in Study 1; shaded subjects were also in Study 2.

Name	Age(Yrs)	Breed	Sex	Status	Bin	Withers	Height (cm) Elbow	Eye	Final Aperture Height
Donald	5.5	Mixed	M	neutered	S	31	12	33	29
Aspen	9.2	Siberian Husky	F	spayed	L	60	28	66	18
Maki	4.5	Chihuahua	M	neutered	XS	22	9	22	8
Charley	7.3	Mini Poodle	F	spayed	M	40.5	20.5	52.5	n/a
Ice Cream Louie	2.7	Mixed	M	neutered	M	48	23	50	16
Morticia	5.9	Greyhound	F	spayed	L	67.5	38	76	61.5
Molly	6.5	Mixed	F	spayed	S	36	20	45	15
Roland	7.6	Yorkshire Terrier	F	spayed	XS	22	8	28	12
Sammy	3.0	Labrador retriever	M	neutered	L	56	28	68	20
Layla	8.1	Mixed	F	spayed	M	46	23	52	16
Enzo	1.3	Mixed	M	neutered	S	--	--	--	10
Jackson	11.1	Mixed	M	neutered	M	53	25	60	23
Wigs	3.0	Springer Spaniel	M	neutered	M	53	27	64	16
Wally	5.8	Bearded Collie	M	neutered	L	58	28	63	28
Maloney	2.6	Mixed	M	neutered	M	53	28	62	15
Jesse	6.1	Shi Tzu	M	neutered	S	31	16	37	28
Nemo	4.8	Mixed	M	neutered	S	26	10.5	31	8
Penny	3.6	Labrador retriever	F	spayed	L	57	33	66	21
Artemis	1.7	Border Collie	F	intact	M	45	24	54	15
Bobby	5.6	Labrador retriever	M	neutered	L	61	32	77	21
Lulu	9.9	Yorkshire Terrier	F	spayed	S	26	14	24	8
River	8.8	German Shepherd	F	spayed	L	68	33	72	44
Jameson	7.2	Mixed	M	neutered	M	45	26	54	15
Milo	3.9	Mixed	M	neutered	L	62	30	68	26
Gotham	3.7	Keeshond	F	spayed	M	41	19	52	16
Walter	5.2	Golden retriever	M	neutered	L	65	37	82	23
Amy	7.2	Bull Terrier	F	spayed	M	48	25.5	54	18
Pepper	4.3	Mixed	F	spayed	L	57	32	60	21
Oliver	2.4	Mixed	M	neutered	M	47	19	54	17
Francie	11.3	Chihuahua	F	spayed	XS	25	11	23	13
Buttons	2.9	Mixed	M	neutered	M	40	19	51	10
Bear	5.3	Mixed	M	neutered	L	67	35	70	31
Izzy	6.0	Mixed	M	neutered	S	33	15	39	21
Lia	8.7	Yorkshire Terrier	F	spayed	XS	23	12	29	11
Arrow	3.3	Airedale Terrier	M	neutered	XL	70	37	80	40
Indy	4.4	Labrador retriever	F	spayed	L	54	22	63	18
Gracie	1.8	Yorkshire Terrier	F	spayed	XS	24	8	29	18
Wyatt	2.3	Mixed	M	neutered	M	44	17	48	14
Mango	8.5	Mixed	F	spayed	S	31	16	36	13
Wellington	4.9	Springer Spaniel	M	neutered	M	50	25	64	15
Camuggi	7.3	Miniature poodle	F	spayed	S	34	17	37	13
Ollie	6.3	Labrador retriever	M	intact	L	60	31	68	42
Mara	5.9	Mixed	F	spayed	L	68	35	80	26
Luz	2.8	Mixed	F	spayed	L	65	33	73	35

**Table 2:** Dimensions used to determine the size of the aperture on the first and subsequent trials.

Dog Size	First Trial Aperture Size (Height of Opening)	Second Trial Size (Height of Opening)	Increments by Which Plexiglas Lowered for Subsequent Trials
XS: 14–25.4 cm	1.5 × HW or 30 cm	HW + 2 cm	2 cm
S: 25.5–39.4 cm	1.5 × HW or 48 cm	HW + 3 cm	3 cm
M: 39.5–53.4 cm	1.5 × HW or 70 cm	HW + 5 cm	5 cm
L: 53.5–68 cm	1.5 × HW or 90.5 cm	HW + 6 cm	6 cm
XL: >68.1 cm	1.5 × HW or 108 cm	HW + 7 cm	7 cm

HW—height at withers.

## Data Availability

The data referenced in this manuscript will be made available by the authors, without undue reservation, to any qualified researcher upon request.
